# Pennsylvania Hospital

**Published:** 1849-12

**Authors:** James S. Levick

**Affiliations:** Resident Physician; Pennsylvania Hospital


### PENNSYLVANIA HOSPITAL.—*Surgical Wards.—Service of* DR. FOX.

*Cases discharged from September 15th to November 1st,* 1849.

| | Cured. | By request. | Died. |
|---|---|---|---|
| Abscess, - - - - - - | 3 | 0 | 0 |
| Burns and Scalds, - - - - | 6 | 0 | 1 |
| Calculus, . - - - - - | 0 | 1† | 0 |
| Caries of finger, - - - | 1* | 0 | 0 |
| Concussion of spine, - - - | 1 | 0 | 0 |
| Contusion, - - - - - | 1 | 0 | 0 |
| Diseased bladder, - - - | 1 | 0 | 0 |
| Disease of ear, - - - - - | 0 | 1 | 0 |
| Do. knee joint, - - - | 2 | 0 | 0 |
| Do. spine, - - - - - | 0 | 1 | 0 |
| Dislocation of humerus, - - | 2 | 0 | 0 |
| Fistula in ano, - - - - - | 1 | 0 | 0 |
| Do. perineo - - - - - | 0 | 1 | 0 |
| Fractures 37, simple 21, viz.: | | | |
| Clavicle, - - - - - | 3 | 0 | 0 |
| Fore arm, - - - - - | 6 | 1† | 0 |
| Jaw, - - - - - - | 0 | 1 | 0 |
| Knee, - - - - - - | 1 | 0 | 0 |
| Leg, - - - - - - | 1 | 0 | 0 |
| Nose, - - - - - - | 1 | 0 | 0 |
| Rib, - - - - - - | 0 | 1 | 0 |
| Skull, - - - - - - | 0 | 0 | 1‡ |
| Spine, - - - - - - | 0 | 0 | 1 |
| Thigh, - - - - - - | 5 | 0 | 0 |

* By amputation. † Eloped. ‡ At base of skull.

| | Cured. | By request. | Died. |
|---|---|---|---|
| Fractures, compound, 16 viz.: | | | |
| Ankle, - - - - - | 0 | 0 | 1‡ |
| Arm, - - - - - - | 2§ | 0 | 0 |
| Finger, - - - - - - | 1 | 0 | 0 |
| Foot, - - - - - - | 1 | 0 | 0 |
| Forearm, - - - - - - | 0 | 0 | 1‖ |
| Jaw (upper) - - - - - | 0 | 1† | 0 |
| Leg, - - - - - - | 1* | 1 | 1 |
| Skull, - - - - - - | 2 | 1 | 0 |
| Thigh, - - - - - - | 1 | 0 | 0 |
| Gonorrhœa, - - - - - | 5 | 1 | 0 |
| Hemorrhoids, - - - | 1 | 0 | 0 |
| Inflamed hand, - - - - - | 2 | 0 | 0 |
| Do. knee, - - - - - | 1 | 0 | 0 |
| Induration of leg, - - - - | 1 | 0 | 0 |
| Injury of spine, - - - - | 3 | 0 | 0 |
| Iritis, - - - - - - - | 2 | 0 | 0 |
| Necrosis, - - - - - - | 2 | 0 | 0 |
| Orchitis, - - - - - - | 1 | 2 | 0 |
| Paraphymosis, - - - - - | 1 | 0 | 0 |
| Paronychia, - - - - - | 1 | 0 | 0 |
| Retention of urine, - - - - | 4 | 0 | 0 |
| Rheumatic ophthalmia - - | 0 | 1 | 0 |
| Rupture of bladder, - - - | 0 | 0 | 1 |
| Sprain, - - - - - - | 2 | 0 | 0 |
| Stricture of urethra, - - - | 2 | 0 | 0 |
| Syphilis, - - - - - | 7 | 0 | 0 |
| Ulcers, - - - - - - | 5 | 0 | 0 |
| Wounds 26, viz.: | | | |
| Gunshot, - - - - - | 1 | 0 | 3 |
| Incised, - - - - - | 4 | 0 | 0 |
| Lacerated, - - - - - | 9 | 4 | 0 |
| Penetrating, - - - - | 2 | 1 | 0 |
| Punctured, - - - - - | 1 | 0 | 0 |
| | 99 | 20 | 10 |

Of the cases recorded as discharged "by request," many were convalescent, and most, if not all, relieved. It not unfrequently happens that individuals are brought to the hospital, who, as heads of families, can be but illy spared from their homes, and who are removed by their friends as soon as they are able to leave their beds. To sailors the restraints of a hospital soon become irksome, and many leave while under treatment. These, on the house record, are placed in the above category, and as such are here transferred.

*Burns.*—During the last two months, several cases of extensive burns and scalds have been treated in the hospital. In one of

* By amputation. † Eloped. § One by amputation. ‡ Of mania a potu. ‖ Of Tetanus.

these, an Italian boy, aged 10 years, the extent of the injury was such as to furnish a most unfavorable prognosis. He had fallen from the deck of a vessel into a pot of boiling water, and, overturning it in the fall, parts of his thorax, the upper extremities, a large portion of the abdomen, and the anterior surfaces of each thigh were shockingly scalded. When brought to the house, his pulse was feeble, and symptoms of approaching collapse presented. Under the application of warmth and slight stimulation he subsequently reacted. Lime water and linseed oil were at first applied to the parts, after which poultices of flaxseed meal were used. On the fourth day after the accident, the appearance of the parts was very unfavorable, and the pulse of the patient had again fallen. After the sloughs had separated, the irritation of the abraded surfaces and the profuse discharge of the ulcers on the thighs and abdomen rendered necessary the exhibition of sulphate of morphia, and of quinia in considerable doses. During an entire fortnight gr. i. of the sulphate of quinia and gr. $\frac{1}{4}$ of sulphate of morphia were given four times daily. Essence of beef and wine whey, of each f℥.ss. every half hour, were also prescribed.

After the sloughs had entirely separated and the discharge lessened, the ulcers were dressed alternately with the cerates of the sub-acetate of lead, and of the carbonate of zinc, under which they healed kindly, and the boy left the hospital well, nine weeks after his admission. In another case recently discharged, the burn was the result of the explosion of a vessel containing boiling alcohol. In this the ulcers, though superficial, involved two-thirds of the thorax, both anteriorly and posteriorly, and nearly the whole of the left arm and forearm. Opiates and quinia were freely given, and the patient recovered rapidly. In both the preceding cases, as well as in another, the result of burn from oil of turpentine, and in a fourth from boiling pitch, where the parts were so injured as to slough, the application of light flaxseed meal poultices appeared particularly grateful to the patient, and very much accelerated the separation of the disorganized tissue.

Where the burn is superficial, the usual practice of the house is to apply lint wetted with equal parts of lime water and olive or linseed oil. This is covered by oil silk, over which carded cotton is laid and retained in its place by a roller. That this domestic remedy facilitates the recovery (probably by more effectually shielding the part from the action of the air) seemed confirmed in two cases.

*Gunshot Wounds.*—There were twenty-five persons brought to the hospital; on the night of the 9th and morning of the 10th of October. Eight only of this number were found sufficiently injured to remain in the house. In the majority the wounds had been made by small shot, which had merely penetrated the skin or superficial muscles, and which were readily removed by the forceps, a counteropening being rarely necessary. In one or two instances, minute pieces of brick appeared to have been discharged.

Of the eight who remained, two were moribund when brought to the hospital, one has since died of gangrene, one has been discharged cured, and four remain, who are rapidly convalescing. The first of these, a colored boy aged 16 years, had been shot in the head by a musket ball. There was a large external wound, and a considerable amount of cerebral substance was found to have escaped. He died in less than an hour.

*On Inspection*, the ball was found to have entered the skull a short distance below the parietal protuberance of the right side—to have passed directly through the brain, and to have made its exit at a corresponding part of the left parietal bone, having, in its passage, completely shattered the skull. The second, an Irishman aged 20 years, received his injury while looking from the second story window of a house in the vicinity of the fire. When brought to the house, six hours subsequently, he was found to have been shot in the right temple, the slug having passed into the brain, portions of which were seen about the wound. His condition was one of almost entire stupor; pulse feeble and oppressed, extremities cold, with some paralysis. A piece of wet lint was laid over the wound, heat and sinapisms were applied to the extremities, but, as was expected, with no appreciable benefit. He died at 4, P. M. A *post mortem* examination was made on the following day. No wound could be found on the body, and but one—that of the right temple—elsewhere. The calvaria having been removed, was found perforated at a point corresponding with the external wound.

While examining its inner surface, a small clot was noticed adherent to the bone about an inch to the left of the median line, and two inches in advance of the lambdoidal suture, corresponding with an irregular opening in the dura mater. The coagulum having been removed, no injury of the superimposed bone could be found. A probe was then carefully introduced into the opening in the dura mater, and passed downwards until it came in contact with a foreign body. The membrane was then divided, and the cerebral mass lifted out, in doing which a medium size slug fell from the inferior part of the brain. A small coagulum was subsequently found resting upon the base of the skull. The entire tract was next laid open, and the slug was found to have passed somewhat diagonally through the upper part of the right hemisphere, crossing the median line, to have struck the inner table of the parietal bone at the point designated by the clot, thence to have been deflected through the posterior part of the left hemisphere down to the base of the brain, having in its primary course passed above the right lateral ventricle, and in its diverted course posterior to that of the left side, leaving undisturbed the integrity of each.

The third, a delicate young man, received a musket ball in the leg, a short distance below the head of he tibia. When brought to the house he was faint from loss of blood, which however, appeared to be exclusively venous. The ball was found to have fractured the fibula, but so far as could be ascertained the tibia was uninjured. Hemorrhage was arrested by applying a small piece of lint over the wound, the limb was carefully placed in a fracture box, and the cold water dressing applied. Small doses of sulphate of morphia were occasionally exhibited. Although very weak, the patient did favorably until the third day following, when symptoms of gangrene of the leg manifested themselves. Stimulating poultices were at once applied to the part; tonics, porter, and a generous diet given, but without arresting the progress of the disease. His extreme debility would have prevented amputation had it been otherwise deemed proper. The mortification rapidly advanced, tetanoid spasms supervened, and the patient died on the 23d inst., thirteen days after the receipt of the injury. No autopsy was obtained. In this case it is not impossible that the anterior tibial, or what is more probable the popliteal artery, may have been so bruised by the ball as to have cut off the supply of blood from the parts below it.

The fourth, a fireman, aged 30, was shot in the right breast. The missile, which could not be felt, but which from the appearance of the wound was probably a small slug, passed obliquely upwards and outwards towards the axilla, and appeared to have buried itself in the pectoralis major muscle. There was no evidence of injured lung, the physical signs were perfectly normal during the whole course of his treatment, and notwithstanding the extreme alarm of the patient (a large muscular man) no unfavorable symptoms presented. Rest and the water dressing constituted his treatment, and he left the house apparently well fifteen days after his admission.

Of the four who remain, two are colored men, in one of whom a small slug passed into the eye through the lower lid, immediately below the lachrymal caruncle. The effusion and inflammation which followed the injury, readily yielded to prompt antiphlogistic treatment, but the patient is recovering with complete amaurosis of the left eye. In the second, a musket ball had passed through the left lung, grazing the pericardium, and was found lying on the posterior part of the thorax under the skin, from which it was removed by a slight incision. This patient, though convalescent is still under treatment.

Two firemen also remain, one of whom received a musket ball in the fleshy part of the thigh. Entering somewhat posteriorly, it passed upwards and outwards without reaching the bone. Under rest and the water dressing, he has rapidly improved ; the tract of the ball having sloughed is now granulating, and he will be discharged in a few days. In the last, a musket ball entered the leg at the inner margin of the spine of the tibia, about two inches below the head of the bone, passed obliquely downwards through the interosseous space without injuring either bone, and was found pressing upon the skin of the calf of the leg, from which it was released by a slight counteropening. There was but little hemorrhage at the time of the accident, and the patient did favorably for several days. On the separation of the slough he was awakened by a profuse discharge of blood, and lost about twelve ounces before it was arrested. The bleeding was stopped by pressure, since which there have been two slight hemorrhages which have been easily checked. The part is now rapidly filling up, and the man will soon leave. In this instance the loss of blood was probably from one of the smaller vessels, but in each the escape from fracture of the bone was truly remarkable.

*Rupture of the Bladder.*—An English weaver, aged 40 years, was brought to the hospital September 14th, having received a serious injury on the previous night. His condition indicated extreme suffering, his face pale and anxious, respiration hurried, pulse feeble, and frequent. There appeared to be no marked paralysis, but from pain or debility the patient was unable to walk. His prostrate condition rendered it difficult to ascertain the manner in which the injury had been received, but so far as could at the time and subsequently be learned, it appeared, that having been left for the night in the upper story of a building he had become alarmed, and in the dark had walked through an open hatchway, alighting directly on his buttocks, one or two stories below. No injury existing in his chest, his abdomen was next examined. This presented no marks of external violence but was found distended and exquisitely painful on pressure, especially in the hypogastric region. As no water had been passed since the accident, a silver catheter was introduced, and a large quantity of very bloody urine drawn off; after which the patient appeared somewhat relieved. On the following morning he was found extremely feeble, suffering much from abdominal tenderness, with the anxious countenance and hurried respiration of the previous day, to which was added great gastric distress, and occasional vomiting of a yellowish watery fluid. The catheter was again introduced and a small amount of urine, not more than two fluid ounces, but natural in appearance, was removed. The diagnosis was somewhat obscure but upon summing up the symptoms, rupture of the bladder was suspected to have occurred. The manner in which the injury had been received, the tenderness in the hypogastric region, the sudden and extreme prostration, the removal in the first instance of so large an amount of bloody urine and the small quantity withdrawn in the subsequent use of the catheter, added to which the absence of fracture of the vertebræ and of paralysis of the lower extremities, seemed to justify this opinion. He remained in this condition for a few days, had frequent vomitings and continued abdominal pain, pulse thread-like, face pale and bathed in sweat. Wine whey and essence of beef were freely given, anodyne fomentations applied to the abdomen, but the patient died on the evening of the 17th. A *post-mortem* examination was made on the following morning. The abdomen having been opened was found to contain several ounces of urine, and the bladder was found ruptured at its superior fundus, very slightly in advance of its median line. A probe introduced through the urethra passed without difficulty through the opening in the viscus. The parts in the vicinity of the bladder were somewhat softened, as if in a state of incipient decomposition; there was no evidence of inflammation, the patient's system probably having at no time after the injury been possessed of vitality sufficient for such a result. The rent was transverse, and admitted three fingers. No other injury could be discovered.

It is much to be regretted that the statements of patients as to the manner in which their injuries are received are so frequently vague and obscure. In order that reliable inferences should be drawn from any case, these statements should be critically correct. In the above instance I was at first disposed to believe that the abdomen had been struck in the fall, but the patient was positive that such was not the case, and this was confirmed by subsequently finding the lower part of the back considerably discolored by bruises. There is little doubt that the bladder was full of urine at the time of the accident, as the man had been confined to the room during the night as a punishment for some neglect of duty. That the secretion of the kidneys is increased by the emotion of fear, is well known.

JAMES S. LEVICK,
Resident Physician.

*Pennsylvania Hospital, November 1st,* 1849.

